# Seed dispersal as a search strategy: dynamic and fragmented landscapes select for multi-scale movement strategies in plants

**DOI:** 10.1186/s40462-020-00239-1

**Published:** 2021-01-29

**Authors:** Jelle Treep, Monique de Jager, Frederic Bartumeus, Merel B. Soons

**Affiliations:** 1 Ecology & Biodiversity group, Department of Biology, Utrecht University, Padualaan 8, 3584 CH Utrecht, The Netherlands; 2 Department of Animal Ecology, Netherlands Institute of Ecology (NIOO-KNAW), Droevendaalsesteeg 10, 6708 PB Wageningen, The Netherlands; 3 Centre d’Estudis Avançats de Blanes (CEAB-CSIC), 17300 Girona, Spain; 4 CREAF, Cerdanyola del Vallès, 08193 Barcelona, Spain; 5 ICREA, Pg Lluís Companys 23, 08010 Barcelona, Spain

**Keywords:** Lévy flight, Lévy walk, Long-distance dispersal, Multi-scale seed dispersal, Plant movement strategies, Short-distance dispersal, Stochastic simulations, Modelling

## Abstract

**Background:**

Plant dispersal is a critical factor driving ecological responses to global changes. Knowledge on the mechanisms of dispersal is rapidly advancing, but selective pressures responsible for the evolution of dispersal strategies remain elusive. Recent advances in animal movement ecology identified general strategies that may optimize efficiency in animal searches for food or habitat. Here we explore the potential for evolution of similar general movement strategies for plants.

**Methods:**

We propose that seed dispersal in plants can be viewed as a strategic search for suitable habitat, where the probability of finding such locations has been optimized through evolution of appropriate dispersal kernels. Using model simulations, we demonstrate how dispersal strategies can optimize key dispersal trade-offs between finding habitat, avoiding kin competition, and colonizing new patches. These trade-offs depend strongly on the landscape, resulting in a tight link between optimal dispersal strategy and spatiotemporal habitat distribution.

**Results:**

Our findings reveal that multi-scale seed dispersal strategies that combine a broad range of dispersal scales, including Lévy-like dispersal, are optimal across a wide range of dynamic and patchy landscapes. At the extremes, static and patchy landscapes select for dispersal strategies dominated by short distances, while uniform and highly unpredictable landscapes both select for dispersal strategies dominated by long distances.

**Conclusions:**

By viewing plant seed dispersal as a strategic search for suitable habitat, we provide a reference framework for the analysis of plant dispersal data. Consideration of the entire dispersal kernel, including distances across the full range of scales, is key. This reference framework helps identify plant species’ dispersal strategies, the evolutionary forces determining these strategies and their ecological consequences, such as a potential mismatch between plant dispersal strategy and altered spatiotemporal habitat dynamics due to land use change. Our perspective opens up directions for future studies, including exploration of composite search behaviour and ‘informed searches’ in plant species with directed dispersal.

**Supplementary Information:**

The online version contains supplementary material available at 10.1186/s40462-020-00239-1.

## Background

Dispersal plays a crucial role in the population dynamics and ecological interactions of plant species. In light of ongoing habitat fragmentation and climate change, dispersal is a particularly critical determinant of local, regional, and global plant species survival [1–4]. This realisation has elevated plant dispersal to a research priority in the last decades. Quantitative information on plant species’ dispersal distance distributions (or ‘dispersal kernels’) is needed for adequate species management, as dispersal kernels determine colonization probabilities, restoration success, and speeds of range expansions and invasions. Indeed, significant progress has been made in understanding how mechanisms of seed dispersal determine seed dispersal kernels [5–8], and how these may be affected by global changes [1, 9–11]. However, the selective pressures responsible for the evolution of dispersal strategies remain elusive.

Excellent overviews of studies on the evolution of dispersal in plants and animals are given in Ronce [12] and Duputié and Massol [13]. For plants, a long line of research has developed on how (kin) competition, facilitation, inbreeding, and density dependent mortality translate to selective pressures for dispersal propensity (the tendency of an individual to disperse, e.g. [14–24]. Increasing dispersal propensity, for example, can serve as an effective bet-hedging strategy to deal with spatiotemporal environmental variability [22]. Few studies to date included dispersal kernels in their evolutionary analysis [25–27]. These studies usually focus on a single aspect of dispersal (e.g., long-distance dispersal) or compare dispersal strategies in a relative sense (e.g., how dispersal propensity increases or decreases in response to stress-related factors). However, many of the driving processes act at different spatial scales [13], so that flexibility in the shape of dispersal kernels is required to balance trade-offs imposed by drivers that operate at a broad range of scales. Other moments of the dispersal kernel also play an important role in population dynamics, and define a dispersal strategy, as has been found in analytical and empirical studies in animal dispersal [28–32].

Following these lines, we argue here that for the purpose of investigating plant ecological and evolutionary processes, it is necessary to move beyond dispersal propensity and single-scale views of the dispersal kernel, and instead assess the entire distribution of seed dispersal distances. We propose that plant dispersal strategies evolve as search strategies for suitable habitat, in a way comparable to stochastic searches made by other moving organisms. Reynolds [33] already hypothesized that plants may maximize the likelihood of finding the nearest unoccupied site by adopting a Lévy flight-shaped inverse power-law seed dispersal kernel. We propose that plant species have a wide range of dispersal strategies, that each evolved in search of *all* suitable habitat of the species, and therefore depend strongly on the spatial and temporal distribution of their habitat. Using a theoretical framework inspired by animal movement ecology, we show how recent conceptual developments in analysing animal movement data can advance the field of plant dispersal ecology towards identification of evolutionary drivers and ecological consequences of seed dispersal strategies.

**Fig. 1:**
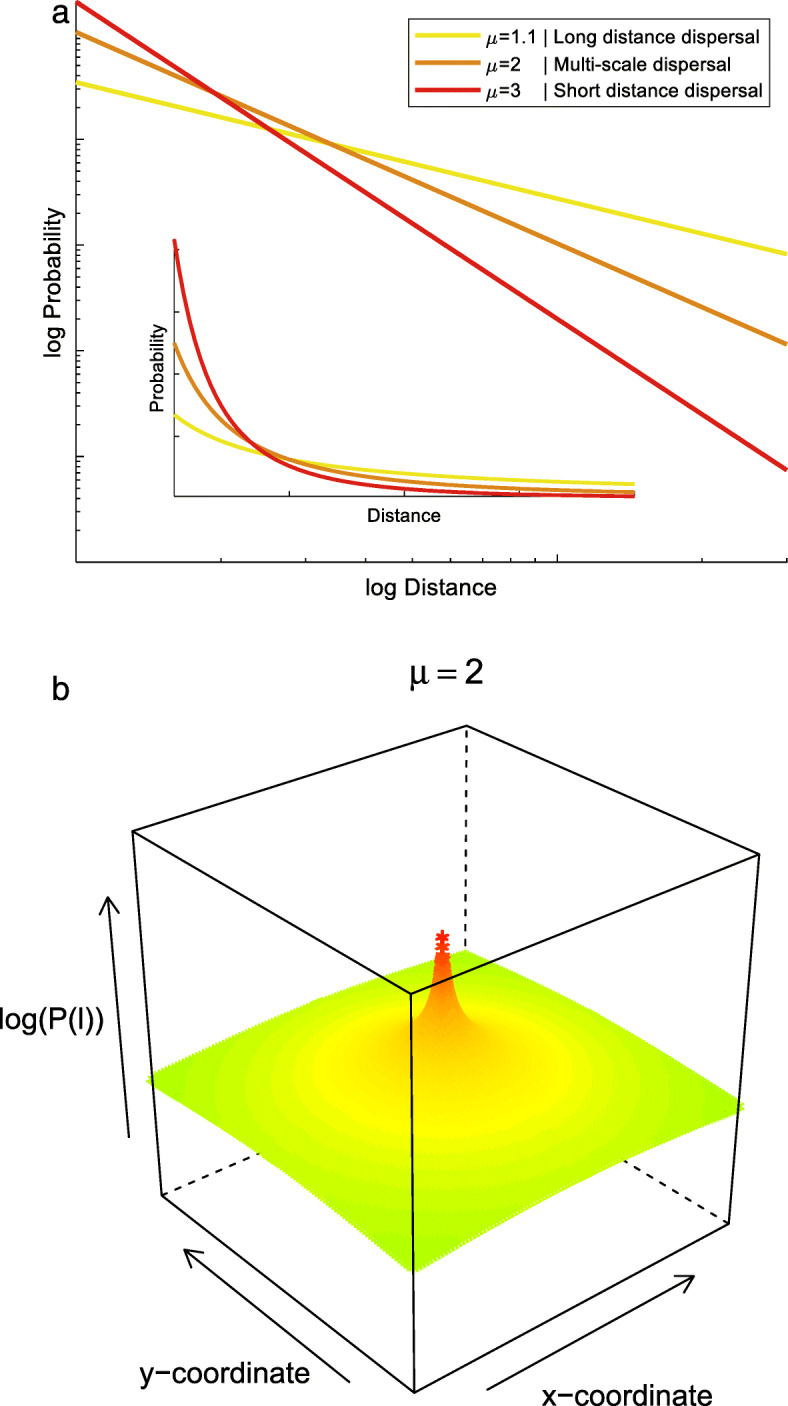
Move length distributions can be described using a Pareto distribution (p(*x*) ~ *x*^-*μ*^; panel **a**). Seed dispersal kernels can be described using a truncated two-dimensional (isotropic) Pareto distribution (Eq. 1; panel **b**). Shape parameter *μ* determines whether dispersal strategies are characterized by a high probability of long-distance dispersal, by balancing long- and short-distance dispersal strategies, or dominated by short-distance dispersal only

In animal movement analysis, movement paths are decomposed into consecutive movement steps of a specific length (move length) that are separated by changes in direction (turns) [34, 35]. Move length distributions can be described by power-law relationships (p(*x*) ~ *x*^-*μ*^; Fig. 1a), where a single scaling parameter governs the relationship between move lengths and their frequency. This scaling parameter *μ* can range from ~ 1 (where steps of all lengths are equally abundant) to > 3 (approximating Brownian motion, where short steps are abundant and long steps are very rare). At an intermediate *μ* ~ 2, many consecutive short-distance movements are alternated with infrequent long-distance movements, producing complex multi-scale movement patterns where many different move lengths occur [35–39]. These complex multi-scale movement strategies are known as Lévy flights or walks [36].

In a random search, the move length distribution that maximizes search efficiency depends on the spatiotemporal distribution of targets being sought (e.g., food, mates [37, 40, 41]). Theoretical studies have shown that various strategies may effectively balance movements across different scales, depending on the spatiotemporal environmental conditions [39–43]. Importantly, this balance is determined by the entire move length distribution. Experimental studies have shown that complex animal movement patterns indeed have intrinsic underlying patterns that optimize random search efficiency, thereby greatly enhancing individual fitness [39, 44–46].

Although these recent developments have significantly advanced our understanding of the evolution and ecology of animal movement [46], the potential for evolution of similar random search strategies has not been explored for plants. We propose that the move length distribution (i.e., seed dispersal distance distribution or dispersal kernel) generated by all the seeds coming from a single plant can be viewed as a movement strategy to search for suitable habitat, or more specifically, suitable sites for germination, establishment and reproduction. As adult plants are otherwise immobile, we expect that there is strong selective pressure on such dispersal kernels.

Here, we provide a theoretical framework that identifies null models for (evolution of) dispersal strategies in plant populations and explores the impact of spatiotemporal landscape structure (habitat fragmentation and patch turnover dynamics) on dispersal evolution, considering dispersal as a search strategy. For our study we used a 2D Pareto kernel, where changes of a single parameter, the scaling exponent, lead to strong changes in the diffusive properties of the kernel. We acknowledge that evolved plant dispersal strategies should match relevant biotic and abiotic factors determining eventual offspring success, such as the spatial structure of density-dependent mortality [47], competition, and facilitation [48, 49]. However, in this study, we first explore the evolution of dispersal strategies in a neutral context where plants are identical except for dispersal strategy. By focussing primarily on dispersal strategy in relation to spatiotemporal habitat distribution, we derive fundamental null models that can serve as the basis for extension in the future to explore effects of additional relevant factors.

## Methods

We developed a straightforward spatiotemporal lattice model to explore the evolution of plant dispersal strategies in landscapes differing in their degree of fragmentation (patch size and inter-patch distance) and landscape dynamics (patch turnover). We excluded any variation in life history traits and competitive interactions between plants; all plants were completely similar entities that only differed in the shape of their dispersal kernels. To explore different dispersal strategies, we used the power-law relationship used in animal search studies, and converted it into a bi-dimensional and normalized dispersal kernel. More specifically, we simulated seed dispersal kernels using a truncated two-dimensional (isotropic) Pareto distribution with discrete values of shape parameter *μ* ranging from 1.1 to 5 (Fig. 1b, Table 1; see Appendix S1 in Supporting Information for derivation of 2D form):

1 $$ p(l)=\frac{1}{2\pi}\left[\frac{2-\mu }{{l_{max}}^{2-\mu }-{l_{min}}^{2-\mu }}\right]{l}^{-\mu } $$

**Table 1:** Parameter ranges used in the different simulation scenarios. Landscape size 512 × 512 grid cells (1 cell can hold one individual)

**Landscape parameters**	**Parameter values**
Patch size [diameter, # grid cells]	1, 2, 4, 8, 16, 32, 64, 128, 256, continuous
Inter-patch distance^a^ [# grid cells]	2, 4, 16, 64, 256, 1024
Patch turnover rate	0, 0.01, 0.05, 0.1, 0.5, 1
**Dispersal parameters**	**Parameter values**
Dispersal kernel (Pareto scale parameter *μ*)	U^b^, 1.1,1.5, 2, 2.5, 3, 3.5, 4, 4.5, 5, MN^c^
Number of seeds per individual^d^	(10), 100, (1000), (10000)

^a^ IPD is calculated as mean free path, see appendix S2

^b^ Alternative (‘benchmark’) kernel 1: uniform dispersal

^c^ Alternative (‘benchmark’) kernel 2: Moore neighbourhood dispersal

^d^ Number of seeds per individual was 100 in main simulations; 10, 1000, 10,000 were used in sensitivity analyses only

where *l*_*min*_ is the minimum distance (radius of a grid cell), *l*_*max*_ is the maximum distance (equal to the domain size) and *μ* is the scaling exponent. The scaling exponent determines the power-law decay of the dispersal kernel. This scaling exponent makes the 2D-Pareto kernel a very convenient tool to explore different dispersal strategies. By changing only one parameter, the kernel can cover a full range of dispersal strategies ranging from very local (short-distance dispersal dominated, *μ* > 3) to non-local (relatively high probability of long-distance dispersal, *μ* → 1) and all scales in between. For *μ* ~ 2 (i.e., canonical multi-scale strategy), this kernel produces a highly heterogeneous distribution of dispersal distances, which slowly decays in frequency from short to long dispersal distances. From search theory we know that the scaling exponent in power law kernels strongly determines the inherent spreading properties (i.e. from Brownian motion to anomalous diffusivity) and effectively trades the sampling effort across scales [35]. This is not always true when considering other kinds of distributions described by a shape and a scale parameter, where the heterogeneity of dispersal scales is not as high as in power laws. Indeed, in power-law kernels, convergence to Gaussian spreading (normal diffusion) is very slow, whereas other distributions may converge to Gaussian spreading very fast.

We compared these 2D-Pareto kernels to ‘benchmark’ kernels or limiting cases on both ends of the spectrum: uniform dispersal across the entire domain (as benchmark for minimum *μ*) and dispersal only to the eight nearest neighbours (Moore neighbourhood) in equal probabilities (as benchmark for maximum *μ*). In each model-run, two plant types with different dispersal kernels competed in a landscape with specified fragmentation and patch turnover characteristics. Plants were initially randomly placed in equal proportions throughout a landscape. For simplicity, plants only produced seeds once per generation and then died (i.e., we simulated semelparous plants). Between generations, the types were redistributed over the landscape following four consecutive steps: 1) dispersal, 2) death, 3) patch turnover, and 4) colonization. The type that remained after multiple generations was assumed to have the better dispersal strategy. We then related these dispersal strategies to three important dispersal metrics that quantify the success of dispersal. A conceptual figure illustrating the model and model details are provided in Appendix S2.

### Simulations

We characterized landscapes by the parameters patch size, inter-patch distance, and patch turnover rate, and determined how evolved dispersal kernels depend on these landscape characteristics. Table 1 shows the setting and parameter ranges used in our simulations.

For each set of parameter combinations, model simulations ran until one of the plant types was outcompeted, i.e. when it occupied less than 5% of all habitat grid cells, while the other type increased to at least 80% of all habitat grid cells or reached a stable equilibrium (no significant decrease over 200 generations). If both types were maintained after 1000 generations, we assigned no ‘winner’ and scored this as no strong selection on dispersal strategy. For each landscape, all possible combinations of dispersal kernels (55 paired simulations) were used to determine the evolutionarily stable strategy (ESS). We repeated each simulation 12 times to test robustness of results. We summarized the outcome of these 12 replicate runs as follows: 1) a clear winner (one population won in at least 11 out of 12 repetitions), 2) extinction of both populations, or 3) no clear winner or no convergence. Most pairwise simulations resulted in a clear winner (at least 11 out of 12 wins), pointing to a clear winning strategy in almost all scenarios. In few cases the 11 out of 12 wins was not reached, namely in highly stochastic scenarios or between two very similar strategies very close to an ESS. Per landscape, these results are presented in pairwise invasibility plots (PIPs, [50]). From each PIP, we extracted the ESS (expressed by parameter *μ*) and used this to identify changes of evolved strategy in relation to patch size, inter-patch distance, and patch turnover rate. We performed robustness tests of our model results to variations in plant seed number (Appendix S3) and our FFT approach (Appendix S4), which both had no significant impact on resulting patterns of ESSs, although a lower seed number (seed production) in some cases leads to no ESS, especially in scenarios with small distant patches and rapid turnover (Fig. S3).

### Dispersal metrics

We expected that, similar to random searches by animals, evolutionarily stable dispersal kernels would adequately balance a complex trade-off across a range of scales conditioned to the landscape configuration (habitat patch size, inter-patch distance and patch turnover). To facilitate interpretation of these trade-offs, we calculated three dispersal metrics that relate to the success of dispersal for each landscape configuration. First, we calculated the success rate of finding habitat (hereafter referred to as ‘habitat encounter’), as the fraction of seeds landing in suitable habitat. Second, we calculated the success rate of avoiding kin competition (hereafter ‘kin avoidance’). Grid cells close to parent plants typically receive a high quantity of seeds (> 1), but only one individual can occupy a cell in the next time step. For each parent, we calculated kin competition using the following steps: I) We selected all grid cells where more than one seed arrived (remember here that we don’t have discrete seeds, so 1.5 seeds in a grid cell is also possible). II) We subtracted 1 seed from the values in these grid cells and summed all remaining (fractions of) seeds. III) We calculated the average number of superfluous seeds for all parents and then normalized the resulting value for the average number of grid cells where more than one seed arrived. Subsequently, we calculated kin avoidance as 1 - kin competition. Kin avoidance has a much stronger spatial signature than conspecific avoidance, which is why we selected this metric to analyse the evolution of dispersal patterns. Third, we determined the success rate of colonizing new patches (hereafter ‘colonization’) as the fraction of dispersed seeds landing in a new patch that emerged due to patch turnover.

## Results

### Evolutionarily stable dispersal strategies are tightly connected to the spatiotemporal distribution of plant habitat

Our simulations show that all types of dispersal strategies (ranging from strategies dominated by short-distance dispersal, to multi-scale dispersal, to long-distance dispersal) can be an ESS, depending on the spatiotemporal distribution of habitat in the landscape. The spatiotemporal distribution of habitat determines the optimal range of scales for dispersal following trade-offs in habitat encounter, kin avoidance and colonization. In general, we found that in the most static and in the most unpredictable landscapes, the two extremes (Moore nearest-neighbour dispersal and uniform dispersal, respectively) are ESS’s. When patch distributions are dynamic and fragmented, the trade-off results in a wide range of multi-scale dispersal strategies (including Lévy-like dispersal) that are tightly connected to the spatiotemporal habitat distribution.

### Short-distance and long-distance dominated dispersal strategies

**Fig. 2:**
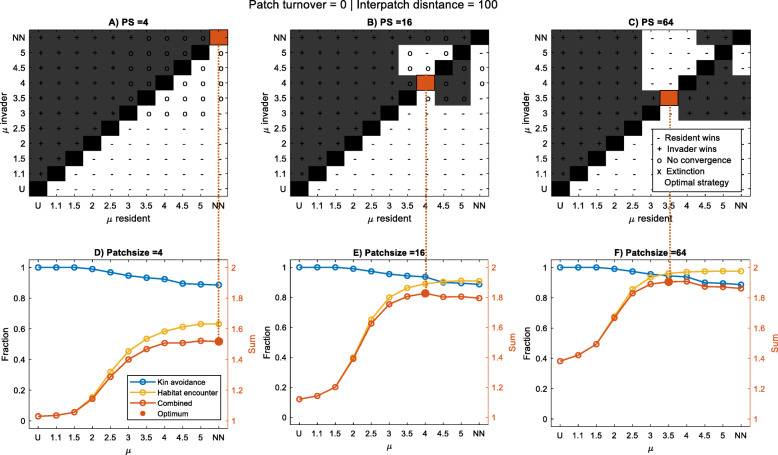
Upper panels (**a-c**): pairwise invasibility plots for dispersal strategies with different values of *μ* (and the extreme cases of uniform and nearest-neighbour dispersal), highlighting the evolutionary stable strategy in red. Grey shading indicates the resident-invader combinations where the invasive type outcompetes the resident type in more situations than vice versa; a ‘+’ indicates that this happened in at least 11 out of 12 replicate runs. No convergence (‘0’) means that either no winner was identified after 1000 generations or no stable outcome was achieved (winning 11 out of 12 replicate runs). ‘X’ indicates extinction of both types. Lower panels (**d-f**): seed fates for dispersal strategies with different values of *μ*, showing on the left y-axis the fractions of seeds that landed in suitable habitat (‘habitat encounter’, yellow line), and the fractions of seeds that avoided kin competition (‘kin avoidance’, blue line). On the right y-axis, the sum of both fractions (‘Combined’, red line) and the optimal *μ* are represented

When the distribution of habitat is patchy in space and static in time, the landscape is highly predictable and movement strategies dominated by short-distance dispersal are evolutionarily stable (Fig. 2a-c). The shape of these short-distance dispersal kernels varies with patch size, with decreasing patch sizes corresponding to shorter dispersal distances (larger *μ*) (Fig. 2a). These movement strategies are essentially driven by the optimization of both habitat encounter and kin avoidance (Fig. 2d-f). With increasing patch size, the edge-to-area ratio decreases and habitat encounter increases accordingly, allowing for strategies with somewhat longer dispersal, which improves kin avoidance. In some situations, when patches are small and inter-patch distances relatively long, several short-distance dispersal strategies can coexist (see the ‘0’s in Fig. 2). In these situations, patches are dominated by one of the plant types within a few generations based on chance and, after that, are almost impossible to be invaded by the other plant type in distant patches as these reach other patches only in low numbers.

**Fig. 3:**
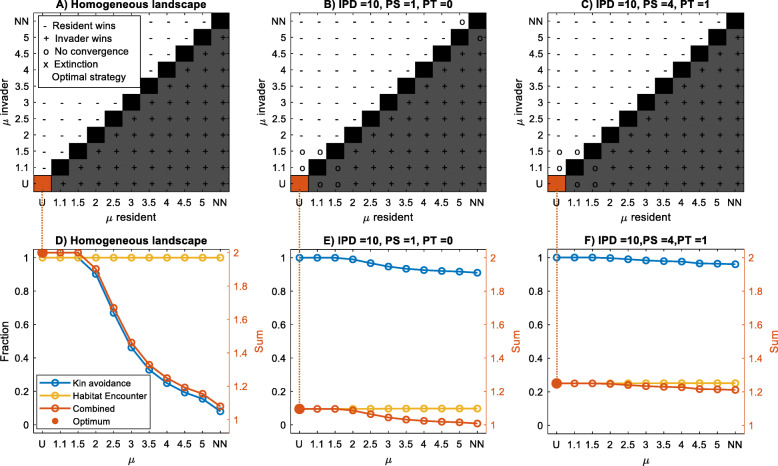
Upper panels (**a-c**): pairwise invasibility plots for dispersal strategies with different values of *μ* (and the extreme cases of uniform and nearest-neighbour dispersal), highlighting the evolutionary stable strategy in red. Grey shading indicates the resident-invader combinations where the invasive type outcompetes the resident type in more situations than vice versa; a ‘+’ indicates that this happened in at least 11 out of 12 replicate runs. No convergence (‘0’) means that either no winner was identified after 1000 generations or no stable outcome was achieved (winning < 11 out of 12 replicate runs). ‘X’ indicates extinction of both types. Lower panels (**d-f**): seed fates for dispersal strategies with different values of *μ*, showing on the left y-axis the fractions of seeds that landed in suitable habitat (‘habitat encounter’, yellow line), and the fractions of seeds that avoided kin competition (‘kin avoidance’, blue line). On the right y-axis, the sum of both fractions (‘Combined’, red line) and optimal *μ* are represented. IPD = Inter-patch distance, PS = patch size, PT = patch turnover rate

When the distribution of habitat is continuous in space (the most predictable scenario) or when the distribution of habitat is unpredictable, either in space (patch size = 1, i.e. patches can hold one individual only) or in time (patch turnover rate = 1), dispersal strategies dominated by long-distances were evolutionarily stable (Fig. 3a-c). In these situations, dispersal strategies are driven exclusively by avoidance of kin competition (Fig. 3d-f). Maximizing habitat encounter does not contribute to selecting better strategies, because habitat encounter is similar for all possible values of *μ*; either because the landscape consists of continuous habitat, or because the habitat distribution is so unpredictable that no *μ* optimizes habitat encounter better than another (Fig. 3d-f).

### Multi-scale dispersal strategies

**Fig. 4:**
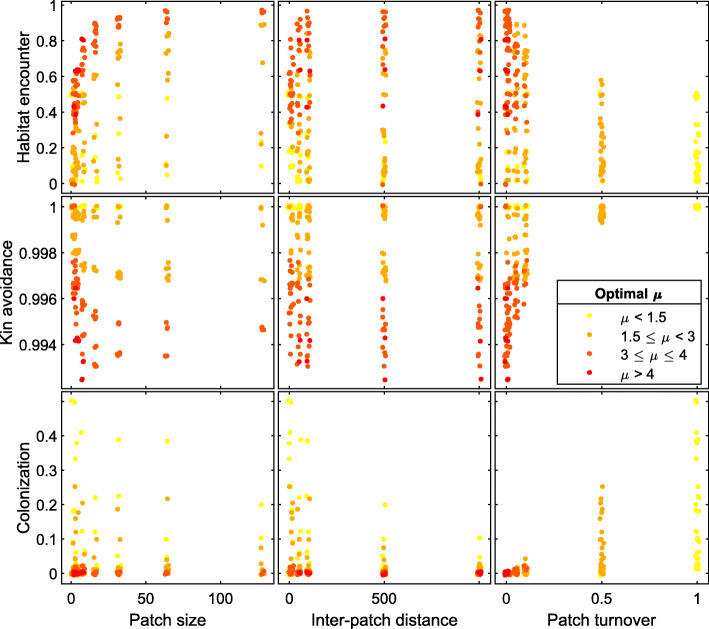
Scatterplots showing the dispersal metrics shaping the dispersal kernel (‘Habitat encounter’, ‘Kin avoidance’, and ‘Colonization’) as a function of landscape parameters (‘Patch size’, ‘Inter-patch distance’, and ‘Patch turnover’). The dots in the scatterplots represent the optimal dispersal kernels

**Fig. 5:**
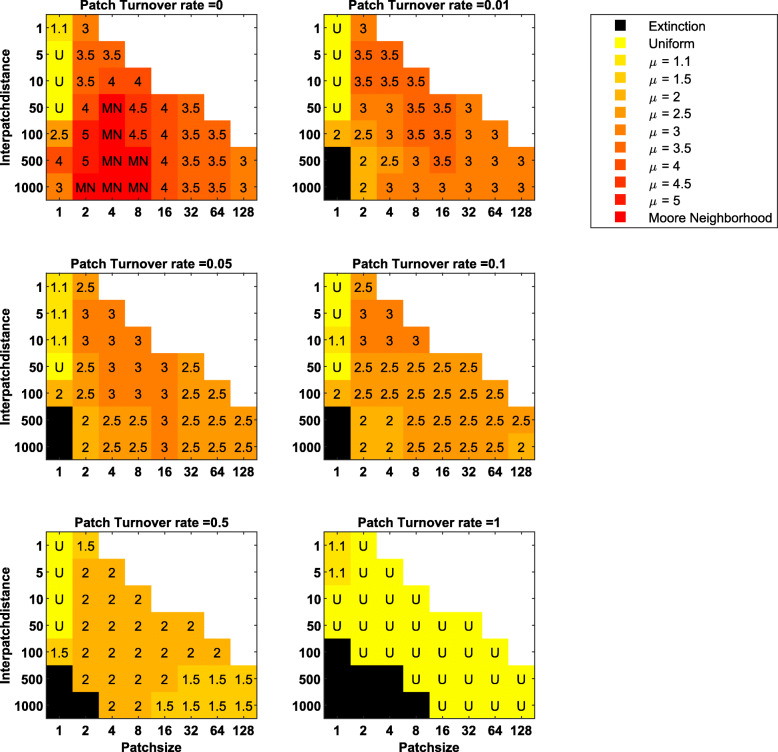
Heat maps showing the evolutionary stable dispersal strategies of all possible pairwise invasibility plots for landscapes with increasing patch turnover rates (from 0, top left, to 1, bottom right), as a function of both patch size and inter-patch distance

In many landscapes, habitat distribution is patchy and dynamic to some extent. In these situations, evolved dispersal strategies are dominated by multi-scale dispersal strategies (e.g., Lévy-like Pareto distributions). These strategies balance local, within-patch dispersal to provide high habitat encounter rates, and non-local dispersal to avoid kin competition and colonize new patches. This balance is driven by all patch distribution characteristics: patch size, inter-patch distance and patch turnover rate (Fig. 4, Appendix S5). The ESS’s for these landscapes, as reflected by *μ*, are most strongly determined by patch turnover rates (Figs. 4, 5, S5), which very strongly increase the need to colonize new patches. Higher patch turnover rates correspond to lower values of *μ*, with *μ* ~ 2 for a wide range of landscapes with patch turnover rates between 0.1 and 1 (Fig. 5). Secondarily, evolutionarily stable dispersal strategies are also driven by the relative importance of colonization of new patches in relation to habitat encounter and kin competition, which decrease with patch size and inter-patch distance (Fig. 4, S5). Under low dynamic conditions, this translates to multi-scale dispersal strategies with more local dispersal when patch sizes are small and inter-patch distances short (as the role of habitat encounter becomes more important, *μ* → 3). Under highly dynamic conditions, this translates to more long-distance dispersal when inter-patch distances are large (as the effects on colonization are strengthened by the role of kin avoidance, *μ* → 1.5; Fig. 4, S5). Mid-range dispersal is an important component of all these strategies.

The short-distance dominated dispersal strategies that we found to be ESS’s in static landscapes with small patch sizes (2–8) and large inter-patch distances (> 50) changed immediately to multi-scale dispersal strategies when patch turnover rate was even slightly larger than zero. Under these conditions, winning the local competition within patches was no longer a stable strategy in the long term. Finally, no dispersal strategy could ensure population survival in some dynamic landscapes with large inter-patch distances. In these cases, the probability of seeds ending up in new habitat was too low to overcome the loss of habitat due to patch turnover.

## Discussion

Studies of animal movement behaviour have identified general optimal movement strategies based upon the spatial distribution of resources [37, 41, 51]. By borrowing intensive-extensive exploration trade-off concepts from animal movement ecology, we show that general optimal dispersal strategies can be identified for plants based purely on the shape of the entire seed dispersal kernel in relation to the spatiotemporal distribution of the plant habitat. Earlier studies have shown how dispersal propensity may evolve in response to landscape structure, cost of dispersal and other (density-dependent) processes [13, 25–27, 52]. Furthermore, studies have shown that other properties describing the shape of the dispersal kernel matter and can be adaptive in certain settings [26, 28, 29, 31]. Here we show that the *entire shape of the dispersal kernel* can be seen as a multi-scale search strategy that needs to balance incentives to disperse over short-distances, long distances and everything in between.

**Fig. 6:**
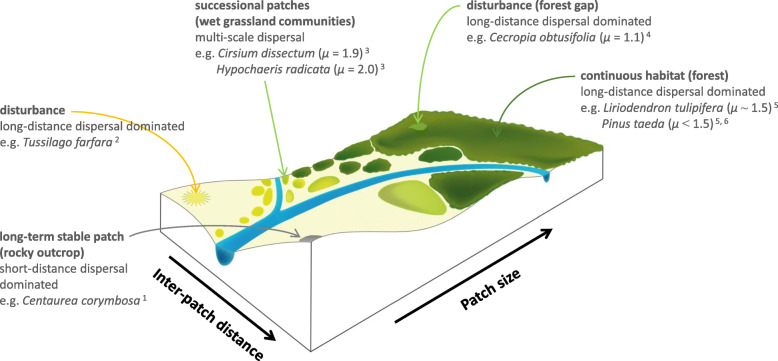
Conceptual diagram showing a range of dynamic (disturbances, successional grassland, forest gaps) and static (rocky outcrops, forests) habitats across gradients in inter-patch distance and patch size. In static, but patchy habitats, short-distance dispersal (*μ* > 3) dominates dispersal strategies. Extreme long-distance dispersal (*μ* → 1) is favoured in both stable, continuous habitats or in extremely unpredictable and dynamic landscapes. In patchy and dynamic habitats, Lévy-like, multi-scale dispersal strategies are optimal, with *μ* correlated to average patch size, inter-patch distance and, most importantly, patch turnover rate. Species examples are given; data from ^1^Colas et al. 1997 [60], ^2^Bakker 1961 [61], ^3^Soons et al. 2005 [62], ^4^Alvarez -Buylla & Martinez-Ramos 1990 [63], ^5^Katul et al. 2005 [64], ^6^Nathan et al. 2002 [65]

The model used in this study is very simple, with individual plants being identical except for differences in dispersal strategies. Competition is not explicitly parameterized in the model, but implicitly there is some form of density dependence due to the role of kin avoidance. Multi-scale dispersal strategies emerge as optimal in a wide range of landscapes even within this simple and straightforward framework, suggesting that they are of importance as a baseline in the natural setting where more processes play a role in spatial population dynamics. The reference framework following from our results is visualised conceptually in Fig. 6. The main hypotheses for real plant data generated from our findings are: (1) In static, but patchy habitats, short-distance dispersal (e.g. *μ* > 3) dominates multi-scale dispersal strategies, due to the importance of optimizing habitat encounter. Particularly when patches are small and inter-patch distances are large, there is a strong selection in favour of extremely short-distance dispersal. (2) In contrast, extreme long-distance dispersal (*μ* → 1, or even uniform dispersal kernels) is favoured in both stable, continuous habitats as well as in unpredictable and dynamic landscapes. These dispersal strategies are driven by avoidance of kin competition and need to colonize newly formed patches. (3) In patchy and dynamic environments, a complex trade-off between finding habitat, avoiding kin competition and colonizing new patches results in multi-scale dispersal strategies with *μ* correlated to average patch size, inter-patch distance and, most importantly, patch turnover rate. Our results suggest that multi-scale kernels similar to Lévy flights (*μ* ~ 2) would be selected for in patchy landscapes with intermediate patch sizes (~ 2 to 100 times the plant size), intermediate inter-patch distances (~ 5 to 100 times the plant size) and relatively high patch turnover rates of around 50% per generation. Some aspects of our findings are in line with well-known patterns observed in plant communities: Plant species in patchy and highly dynamic habitats typically have dispersal strategies dominated by long-distance dispersal and species from patchy but highly static landscapes tend to display predominantly short-distance dispersal that promotes the chance of success in ‘winning the home patch’ [22, 53–55]. Yet, such hypotheses are not trivial. For example, in static but patchy landscapes, short-distance dispersal strategies may rapidly evolve. Colonization has been followed by rapid loss of long-distance dispersal in plants on islands and patches in urban environments [56–58]. Such species are extremely vulnerable to habitat loss and fragmentation, as their dispersal strategy is not adapted to colonizing new areas [59]. With ongoing global change, such dispersal-limited species are under great threat of extinction – an example of such a case is the endemic and highly threatened *Centaurea corymbosa* which is adapted to long term persistent, but isolated rocky outcrops [60].

Some hypotheses generated within our study may appear counterintuitive. For example, species in homogeneous habitats are suggested to have uniform dispersal kernels. This hypothesis would explain why, indeed, many species of large-scale, more or less continuous habitats, such as primary forest [65] and heathlands [66], have adaptations for very long-distance dispersal. Previous studies may have suggested that these adaptations serve to avoid density-dependent mortality close to the parent [16, 67, 68], but this would not explain dispersal over more than a few tens of m (the decay rate of pest-induced mortality, [24]). Our results suggest that selection for kin avoidance may explain these long-distance dispersal syndromes, although escaping density-dependent mortality may be an additional, enforcing factor.

Our analyses also lead to interesting untested hypotheses: species subjected to patchy environments should have multi-scale dispersal strategies that vary in the fatness of their tail in relation to patch size and inter-patch distances, but primarily in relation to patch turnover rates. This means that mid-range scales may become more or less prominent depending on habitat distribution characteristics. Analyses of measured plant dispersal kernels across real landscapes should reveal whether these hypotheses indeed reflect reality. It is, however, difficult to obtain complete dispersal kernels from field measurements, as long-distance dispersal events are extremely difficult to measure and at the same time form a vital component of the dispersal strategy. For wind dispersal, mechanistic models have been developed that simulate complete dispersal kernels (including long-distance dispersal events), and these have withstood tests against field tracking and trapping data (e.g., CELC , [69]; and WALD, [64]). Simulations of tree dispersal kernels using WALD indicate that forest trees such as *Liriodendron tulipifera* in oak-hickory forests, one of the largest and most continuous forest habitats in temperate regions, could have tails with power laws of *μ* ~ 1.5 [64]; species such as *Pinus taeda* are likely to have even fatter tails [65]. These kernels are close to the long-distance dominated dispersal kernels that would be expected for species in continuous habitats. Simulations of wind dispersal using the CELC model for herbs characteristic of patchy and temporary wet grasslands ( [69]; data from [62]) generate dispersal kernels that are best fitted by 2D-Pareto distributions with *μ* ~ 2 (1.9 for *Cirsium dissectum*, 2.0 for *Hypochaeris radicata*). These values match the Lévy-like multi-scale dispersal kernels expected for species in successional, patchy habitats. For species typical of highly disturbed sites, such as *Tussilago farfara* in disturbed open sites, extreme long-distance dispersal has been reported - up to 4000 m in one generation [61], with a roughly estimated *μ* of 0.59 [70]. Another species typical of disturbed sites is *Cecropia obtusifolia*, a pioneer tree colonizing forest gaps. Seed trap data of this species in young forests are best fit by a 2D-Pareto distribution with *μ* = 1.1 (data from [63]). We summarize these first lines of evidence in Fig. 6.

The occurrence of multi-scale dispersal strategies in nature becomes particularly apparent in species that combine multiple dispersal vectors that transport seeds across different scales. For example, *Cakile maritima* produces two different types of seeds that are either dispersed by water or as tumble weed attached to the maternal plant [71]. In other species, the same seed types are intended to be dispersed by different vectors, such as plants with seeds in fleshy fruits (dispersed by a range of animal species with varying dispersal capacities [72],) and wetland plants (dispersed by water or by waterbirds, [73]). Investments in seed morphology to optimize dispersal come at widely varying costs [52], which may also be driven by post-dispersal processes.

We have not included such dispersal costs and trade-offs in seed size, seed number, and dispersal capacity in our simple model in order to facilitate the exploration of optimal dispersal strategies in relation to spatiotemporal habitat distribution. Other trade-offs and costs are expected to alter subsequent population dynamics (e.g. we see that low seed production will not lead to ESS in certain scenarios (Fig. S3)) and future directions would be to include such trade-offs in the model and determine their relative importance relative to spatiotemporal habitat distribution. For similar reasons, we deliberately chose a 2D Pareto kernel, where changes of one parameter, the scaling exponent, lead to radical changes in the diffusive properties of the kernel. Therefore, despite being a very simplified model compared to e.g. dispersal kernels that are described with a shape and scale parameter, we know from search theory, that power-law based kernels can optimally adjust between near and far dispersal scales in different contexts by generating anomalous or enhanced diffusive properties [35], allowing us to explore the possibility to study the adjustment of radically different types of spreading in a wide range of contexts. As a limitation, simplifying the dispersal kernel into one parameter reduces our degrees of freedom to explore the impact of scale and shape parameters separately. In the case of the 2D Pareto kernel bounded by a minimum (*l*_*min*_) and a maximum (*l*_*max*_) spreading distance, the variance and other moments of the distribution can be computed. Importantly these moments depend not only on the scaling exponent but also on the spreading range, *l*_*max*_ acting here as a second parameter worth studying as it influences the variance and other moments. An interesting future avenue in this framework is to analyse how the dispersal range (and not only the scaling exponent) modulates the spreading strategy, and compare the 2D Pareto kernel with other flexible kernels (described by a scale and a shape parameter) to study in more detail which parts of the kernel are modulated by which mechanism. Lowering *l*_*min*_ to below the radius of a patch would allow for within patch dispersal. This could make biological sense and would change the results of especially the patch size is equal to one plant size scenarios.

By analysing seed dispersal as a plant search strategy for finding suitable habitat and using kernels with different scaling behaviour to compare dispersal strategies across different landscape dynamics, we break with the common practice of investigating dispersal propensity or focusing only on a single scale of plant dispersal kernels such as the tail or modal distance. With methodological hurdles to the study of long-distance dispersal being overcome [65, 74], much research has focused on quantifying the tail of the dispersal kernel [5, 74–76]. This has resulted in rapid progress in our understanding of, and ability to predict, the connectivity of plant populations in fragmented landscapes [77, 78] and has helped to explain species’ abilities to track climate change [3, 79] or become invasive [80]. At the same time, other studies have focused on the mode of the dispersal distribution to facilitate cross-species comparisons [81]. The mode represents the distance where most seeds end up, which is a far easier measure and therefore an attractive parameter to study. We however stress that *the entire dispersal kernel* (i.e. all the moments of the distribution) defines the movement strategy of plants, and as such is relevant for local, landscape-scale, and global species survival. Such an integrated approach to plant dispersal has also been advocated in the general ‘movement ecology paradigm’ [82], and an important first step in making large cross-species comparisons of entire dispersal kernels has recently been taken [11]. The simplicity of our approach, which uses a flexible dispersal function parameterized by a single parameter, *μ*, facilitates further comparisons across large numbers of species with widely differing dispersal strategies, while also allowing for the exploration of relations between species’ dispersal strategies and their traits, life history strategy, or habitat characteristics.

As a final point, we hope our framework facilitates plant ecological research to benefit from conceptual advances in animal movement ecology. Promising future directions for plant ecological research include exploring how different costs of dispersal (e.g. due to investments in traits) modify the optimal search strategy (cf. [41]) and examining plant dispersal kernels for the existence of ‘composite walks’, which combine multiple movement types into one dispersal strategy, not necessarily showing scaling properties (cf. [83]). The latter would be relevant in species with dispersal dimorphisms or species using multiple dispersal vectors, as discussed above. Another interesting direction would be to explore to what extent plant searches can be considered as ‘informed searches’. There is a growing body of evidence that plants dispersed by animals, water, and wind utilize ‘directed dispersal’ strategies, in which they use environmental cues or select specific vectors that result in disproportionate arrival of seeds at more suitable sites [84–86]. In a recent study, ‘informed dispersal’ has been suggested as a strategy to escape competition and environmental stress [87]. Thus, future research could explore these strategies in the light of ‘informed searches’ in plants, similar to how animals use cues to guide their search towards suitable sites (cf. [88]). Insights in how these factors shape the evolution of dispersal strategies, and progress in knowledge of dispersal mechanisms, can mutually inspire each other, and thereby improve the understanding and quantification of dispersal in plants.

## Conclusions

Our results clearly show that the full range of dispersal kernels, from extreme long-distance to short distance dispersal, can be adaptive, depending on the spatiotemporal habitat distribution across the landscape. Intermediate landscape dynamics and fragmentation would lead to the most complex and heterogeneous (multi-scale) kernels in terms of seed distributions. These multiple scales reflect an intensive-extensive search trade-off that determines the success rates of habitat encounter, kin avoidance, and colonization of new patches. Our analysis can serve as a framework that generates null hypotheses for dispersal strategies of plant species based on the spatiotemporal distribution of their habitat that can be used to analyse and compare plant dispersal data.

## Supplementary Information

**Additional file 1.**

## Data Availability

The model code and data supporting the results are available on Github: https://github.com/jelletreep/patch-dispersal.

## Abbreviations

2D: Two-dimensional
ESS: Evolutionarily stable strategy
FFT: Fast-fourier transform
IPD: Inter-patch distance
NN: Nearest neighbour
PIP: Pairwise invasibility plot
PS: Patch size
PT: Patch turnover rate
U: Uniform
