# Supplementary material for: Seed dispersal as a search strategy: dynamic and fragmented landscapes select for multi-scale movement strategies in plants
Source: Mov Ecol. 2021 Jan 29;9:4. doi: 10.1186/s40462-020-00239-1 (PMC7845050; doi:10.1186/s40462-020-00239-1)
Supplement: Supplementary file 1 — Additional file 1. [file 40462_2020_239_MOESM1_ESM.docx]

**SUPPORTING INFORMATION**

**Seed dispersal as a search strategy: dynamic and fragmented landscapes select for multi-scale seed dispersal**

**Jelle Treep^1*^, Monique de Jager^1,2^, Frederic Bartumeus^3,4,5^,  Merel B. Soons^1,2,3^**

^1^ Ecology & Biodiversity group, Department of Biology, Utrecht University, Padualaan 8, 3584 CH Utrecht, The Netherlands;

^2^ Department of Animal Ecology, Netherlands Institute of Ecology (NIOO-KNAW), Droevendaalsesteeg 10, 6708 PB Wageningen, The Netherlands;

^3^ Centre d’Estudis Avançats de Blanes (CEAB-CSIC), 17300 Girona, Spain ;

^4^ CREAF, Cerdanyola del Vallès, 08193 Barcelona, Spain;

^5^ ICREA, Pg Lluís Companys 23, 08010 Barcelona, Spain.

***Corresponding author**: Jelle Treep, Ecology & Biodiversity Group, Department of Biology, Utrecht University, Padualaan 8, 3584 CH Utrecht, The Netherlands, hjtreep@gmail.com.

**Contents:**

**Appendix S1: Derivation of the 2D-Pareto kernel. p2**

**Appendix S2:** **Model details and example of model simulations. p3**

**Appendix S3: Sensitivity analysis on seed number. p7**

**Appendix S4:** **Simulations using discrete dispersal events. p9**

**Appendix S5:** **Contributions of habitat encounter, kin avoidance and p11**

**colonization to determining optimal dispersal strategies.**

**Appendix S1:** **Derivation of the 2D-Pareto kernel.**

To derive a truncated 2-D probability density function (PDF) from a 1-D Pareto distribution, the function is normalized by a normalization constant (c). Starting with a 1-D probability density function

$p\left( l \right)= cl^{-\mu}$,

Eq. S1.1

where *l* is distance and µ the scaling exponent, the normalization constant can be derived by taking the integral of equation S1.1 over 360 degrees and from the minimum to the maximum distance. This integral, multiplied with the normalization constant should equal 1;

$$c\int_{0}^{2\pi} d\theta\int_{l_{min}}^{L_{max}} p\left( l \right) dl=1$$

Eq. S1.2

where *L_max_* is the maximum (or truncation) distance and *l_min_* is the minimum distance. Integration of equation S1.2 over 360 degrees and from *l_min_* to *L_max_* yields:

$$2\pi c\left[ \frac{L^{-\mu+2}}{-\mu+2}-\frac{{l_{min}}^{-\mu+2}}{-\mu+2} \right]=1$$

Eq. S1.3

Rewriting for c:

$$c=\frac{1}{2\pi}\left[ \frac{-\mu+2}{L^{-\mu+2}-{l_{min}}^{-\mu+2}} \right]$$

Eq. S1.4

The 2D Pareto kernel is then obtained by combining equation S1.1 and S1.3:

$$p(l)=\frac{1}{2\pi}\left[ \frac{2-\mu}{L^{2-\mu}-{l_{min}}^{2-\mu}} \right]l^{-\mu}$$

Eq. S1.5

Equation S1.5 does not exist when *µ* equals 2. Therefore 2.0001 is used instead of 2.

**Appendix S2:** **Model details and example of model simulations.**

***Lattice***

We constructed a lattice model with a two-dimensional spatial domain of 512 by 512 grid cells. The landscape was simplified to have each grid cell represent either suitable or unsuitable habitat. Landscape configurations were generated randomly using a predefined patch size and inter-patch distance. Patches were circular-shaped and patch size was defined as patch diameter in number of grid cells. We defined a landscape’s inter-patch distance as the average distance from the border of a patch to the border of the nearest patch in all directions. This distance is equal to the mean free path minus the patch radius. Mean free path (*λ*) relates to patch size as follows:

(1)

$$\lambda=\frac{N}{2L^{2}r}$$

where *N* is the number of patches, *L*^2^ the size of the lattice and *r* the patch radius. Patches were randomly placed in the landscape so that they did not overlap, but could potentially adjoin other patches. Each grid cell which was classified as habitat could be occupied by only a single individual from one of the two types.

***Population and landscape dynamics***

1. *Dispersal*

Each individual dispersed 100 seeds to neighbouring cells according to a truncated 2D-Pareto distribution (see Appendix S1 in Supporting Information for derivation of 2D form);

(2)

$$p(l)=\frac{1}{2\pi}\left[ \frac{2-\mu}{{l_{max}}^{2-\mu}-{l_{min}}^{2-\mu}} \right]l^{-\mu}$$

where *l_min_* is the minimum distance (radius of a grid cell), *l_max_* is the maximum distance (equal to the domain size) and *µ* is the scaling exponent. The 2D dispersal kernels were predefined for an individual in the centre of the domain, bounded by the domain boundaries (*l_max_*) and normalized, so that the probability over the entire domain summed to one. In all cases, there was no dispersal to the grid cell of the parent plant (distance = 0). Seeds inherit the dispersal strategy of the parent plant (without mutations). For each landscape, all possible combinations of two dispersal kernels were simulated. The domain was isotropic and had periodic boundaries. Seed dispersal from all individuals of a type was calculated simultaneously by convolution using Fast Fourier Transformations (FFT) (Powell 2002).

1. *Death*

After seed dispersal, all individuals died, thereby resembling semelparous species that have only a single reproductive event in their lifetime.

1. *Patch turnover*

Patch turnover was determined stochastically for each patch using a fixed probability of turnover per patch per time step (each time step equalling the time between two generations). When patch turnover occurred, the patch disappeared and a new patch was randomly placed at a different location in the lattice.

1. *Colonization*

We simulated colonization of empty habitat cells by the dispersed seeds as follows. We calculated the expected number of seeds arriving at a grid cell as the sum of the probability density functions of all individuals of the population (obtained using FFT), multiplied by the number of produced seeds. Using FFT is computationally much more efficient than simulation of discrete dispersal events, however, in doing so, one neglects some of the stochasticity naturally involved in the dispersal process. We re-introduced this stochasticity in the translation of the continuous seed arrival expectations into discrete colonization events (as a grid cell can only hold one individual). First, we determined for both types whether they colonized a certain grid cell. When the expected number of seeds of a type was above 1, the grid cell was colonized by this type. When the expected number was below 1, a random number determined whether the grid cell was colonized by this type or not; $X \sim U\left( 0,1 \right)< p_{i}$, where *p_i_* is the expected number of seeds either from type 1 or 2. This resulted in three possible outcomes: 1) neither types colonized the grid cell, 2) one of the types colonized the grid cell, or 3) both types colonized the grid cell. When both types colonized a grid cell, a second random number (*X ~ U(0,1)*) determined which type would occupy the grid cell in the next time step; $X < {p_{1}}/\left( p_{1}+p_{2} \right)$. An individual of type 1 or 2 occupied the grid cell when *X* = 1 or *X* = 0, respectively.

**Figure S2.1**. The top left panel represents a randomly generated initial landscape for patch size 8 and inter-patch distance 50 (note: for visualization purposes a domain of 128 by 128 grid cells is used here instead of 512). The top right panel shows examples of two 1D dispersal kernels on log-log scale (before transformation to 2D kernels) of the two species competing in a model run. The bottom panel shows the population size of both species as a function of time. In this specific run, the population with a relatively high probability of short distance dispersal (*μ* = 3) increases in size in the first generations. However, due to patch turnover, patches where this population dominates disappear and the better colonizer (*μ* = 2) eventually wins.

**Appendix S3: Sensitivity analysis on seed number.**

We performed a sensitivity analysis to plant seed number, by varying the amount of seeds dispersed per plant several orders of magnitude, representing a realistic range for annual plant species (10, 1000, 10000) (Jakobsson and Eriksson 2000), in ten landscapes with large variation in fragmentation and dynamics (Table S3). We compared the results to the results of the main simulations, where 100 seeds were produced per individual. In general, observed patterns were very similar when comparing pairwise invisibility plots from runs with different seed numbers (Fig. S3). In highly dynamic landscapes and for certain dispersal strategies, low seed numbers resulted in extinction (Table S3). In landscapes with low patch turnover dynamics, optimal *μ* increased slightly with seed number, suggesting slightly stronger selection for habitat encounter.

| **Patch size** | **Inter-patch distance** | **Patch turnover rate** | ***μ_opt_***  ***#10*** | ***μ_opt_***  ***#100*** | | ***μ_opt_***  ***#1000*** | ***μ_opt_***  ***#10000*** |
| --- | --- | --- | --- | --- | --- | --- | --- |
|  |  |  |  | |  |  |  |
| 8 | 50 | 0.1 | 2.5 | | 2.5 | 3 | 3 |
| 2 | 5 | 0.5 | 2 | | 2 | 2 | 2 |
| 1 | 50 | 1 | X | | 1 | 1 | 1 |
| 1 | 10 | 0 | 3 | | 1 | 1 | 1 |
| 128 | 500 | 0 | 3 | | 3 | 3 | 3 |
| 2 | 100 | 0 | 4.5 | | 4.5 | 4 | 4 |
| 4 | 500 | 0.01 | 2.5 | | 2.5 | 3 | 3 |
| 32 | 50 | 0.05 | 2.5 | | 2.5 | 3 | 3 |
| 16 | 500 | 0.5 | 2 | | 2 | 2 | 2 |
| 2 | 5 | 0.01 | 3 | | 3.5 | 3.5 | 3.5 |

**Table S3. Sensitivity analysis, based on 10 selected landscape scenarios, of optimal *μ* (*μ_opt_*) for different values of the parameter ‘number of seeds produced per individual’.**


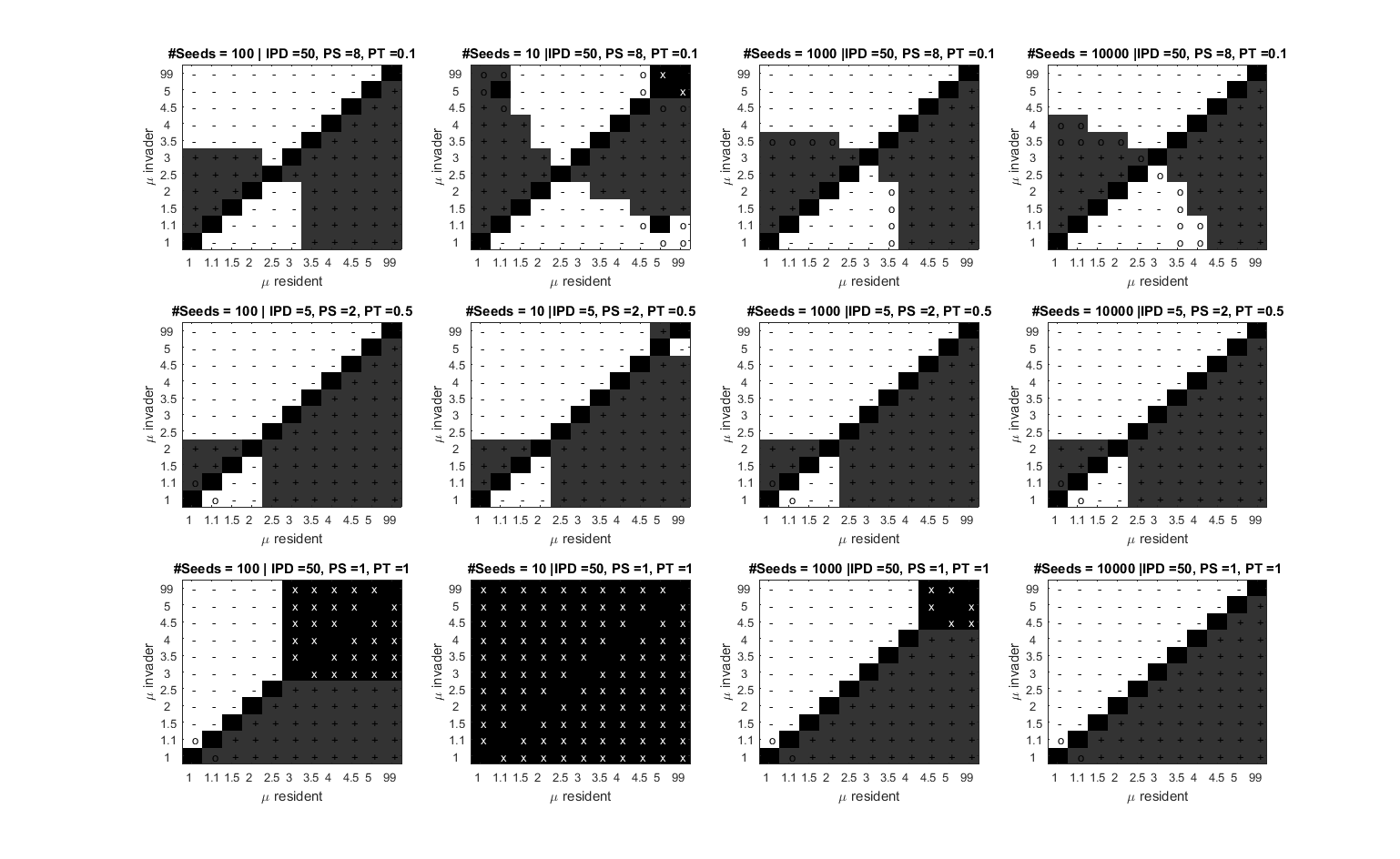

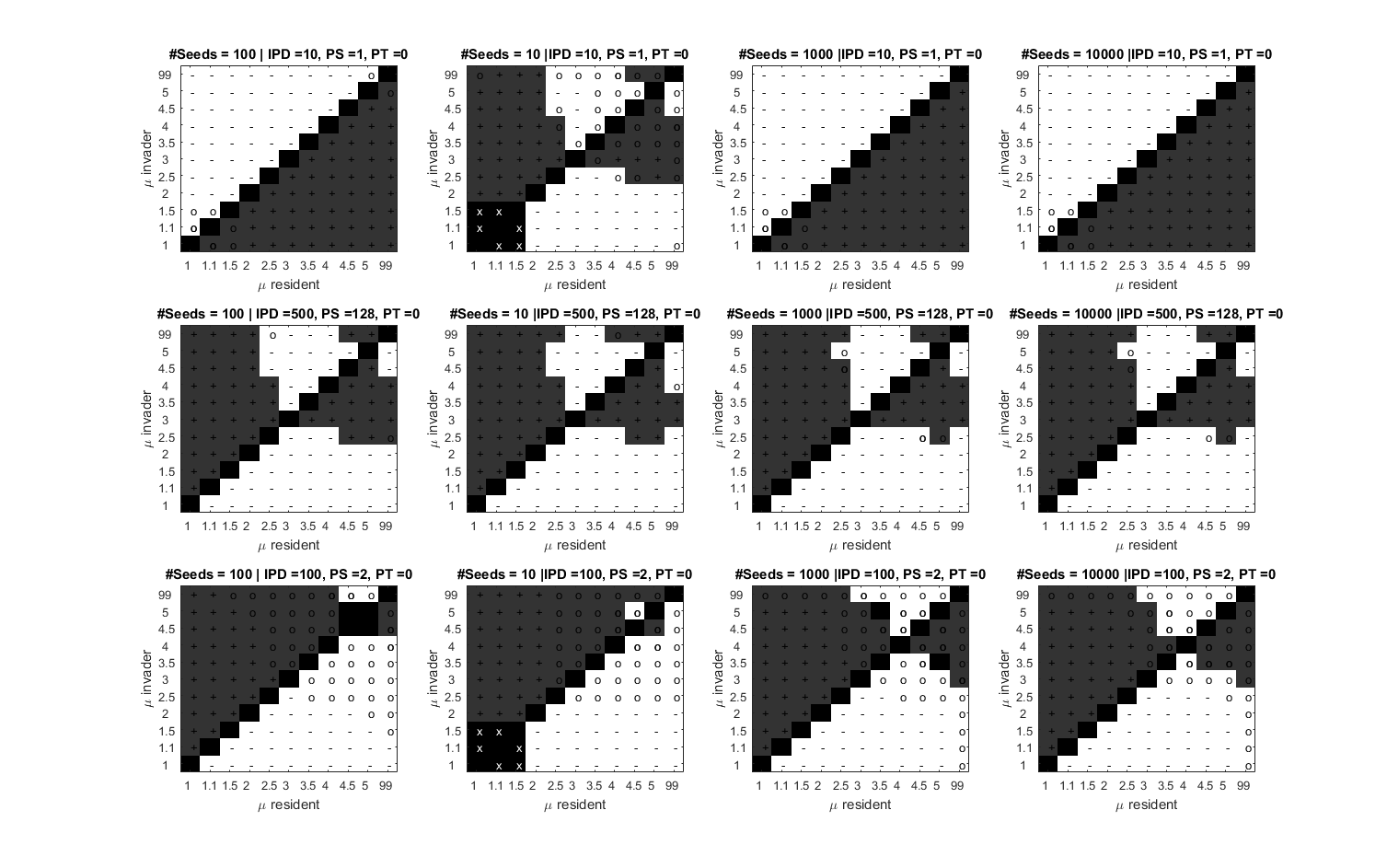


**Figure S3.** Pairwise invasibility plots for 4 out of 10 landscape scenarios that were run in the sensitivity analysis of optimal *μ* (*μ_opt_*) for different values of the parameter ‘number of seeds produced per individual’. Grey shading indicates the resident-invader combinations where the invasive population outcompetes the resident population in more situations than vice versa; a ‘+’ indicates that this happened in at least 11 out of 12 replicate runs. No convergence (‘0’) means that either no winner was identified after 1000 generations or no stable outcome was achieved (winning < 11 out of 12 replicate runs). ‘X’ indicates extinction of both populations.

**Appendix S4:** **Simulations using discrete dispersal events.**

To improve computational efficiency, we used convolution via Fast Fourier Transformation to calculate dispersal for all individuals simultaneously, thereby neglecting some of the stochasticity that is involved in discrete dispersal events. To test whether this simplification affected the competition dynamics considerably, we ran model simulations using discrete dispersal events for 2 landscapes and compared them to the model simulations using FFT. In these simulations, we sampled dispersal events of individual seeds from the 2D-Pareto kernel, and summed the total amount of arrived seeds in each grid cell instead of summing up FFT-derived probabilities. We found only small differences when comparing the results of the two models (Fig. S4). Stochasticity that is present in both models is likely to have caused the slight deviations that are seen around *μ* = 2.5 and *μ =* 3 in the top row. As the FFT-models were computationally much faster, we therefore only present the results of the FFT models.

**
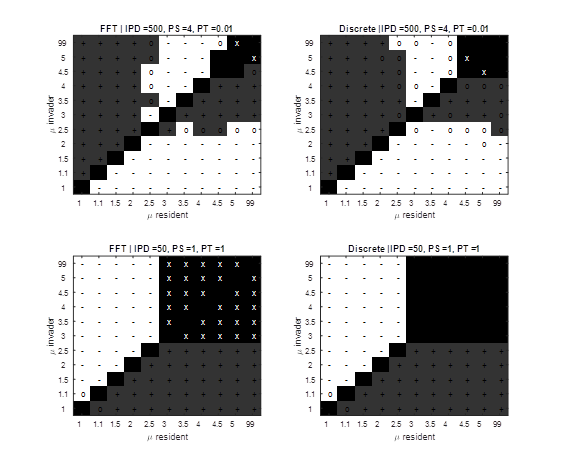
**

**Figure S4.** Pairwise invasibility plots for 2 landscape scenarios for the main model using FFT (left panels) compared with a model with discrete dispersal events (right panels). Grey shading indicates the resident-invader combinations where the invasive population outcompetes the resident population in more situations than vice versa; a ‘+’ indicates that this happened in at least 11 out of 12 replicate runs. No convergence (‘0’) means that either no winner was identified after 1000 generations or no stable outcome was achieved (winning < 11 out of 12 replicate runs). ‘X’ indicates extinction of both populations.

**Appendix S5:** **Contributions of habitat encounter, kin avoidance and colonization to determining optimal dispersal strategies.**

The absolute rates of habitat encounter, kin avoidance, and colonization that are achieved in the optimal dispersal strategies correlate strongly with habitat patch size and patch turnover (and to a lesser extent inter-patch distance, not shown here) (Fig. S5a). With increasing patch size, more habitat can be found close to the parent plant, which increases habitat encounter and decreases kin avoidance. With increasing patch turnover rate, habitat is less predictable which decreases habitat encounter and increases kin avoidance. As more new patches are formed, colonization rate also strongly increases with patch turnover rate. However, these absolute rates are not fully comparable and so do not fully clarify the importance of each of the forces in determining optimal strategies. By normalization of the absolute rates, we obtain better insight into whether the optimal strategy is driven by habitat encounter, kin avoidance, and/or colonization. This normalization is obtained for each landscape separately by:

P_norm_ = (P_opt_-P_min_)/(P_max_-P_min_), Eq. S5.1

where P represents the dispersal metric in question (habitat encounter, kin avoidance or colonization). A value of 1 means that the highest value is selected in the optimal strategy, and 0 means the lowest value is selected. A high value can thus be interpreted as the dispersal metric being a very important factor for that landscape scenario, and 0 as relatively unimportant (e.g., due to a very narrow range). Normalized rates of habitat encounter, kin avoidance, and colonization show that static landscapes (no patch turnover) select for a maximization of habitat encounter, whereas highly fragmented (patch size = 1) and highly dynamic (patch turnover = 1) landscapes select for a maximization of kin avoidance. In all other landscapes a complex trade-off balances habitat encounter, kin avoidance and colonization. Here, all three factors play a role and colonization becomes increasingly important with increasing patch turnover and decreasing patch size.

**
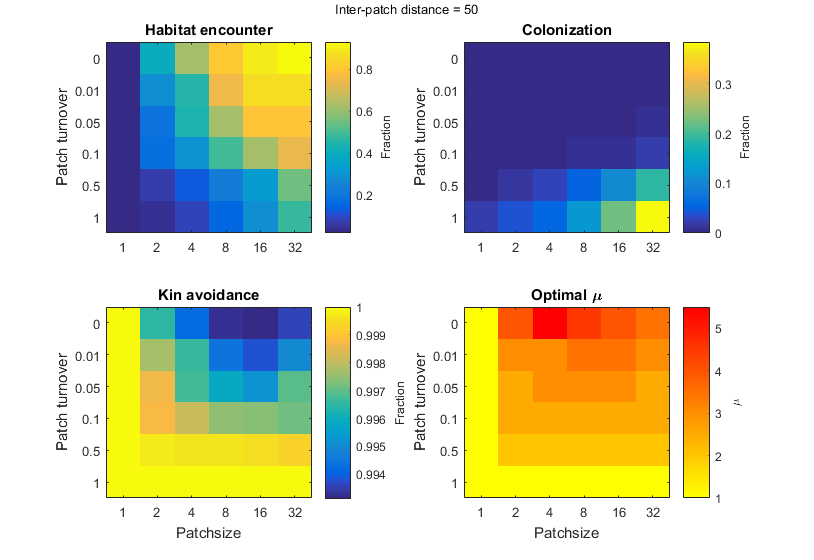
**

**Figure S5a**. Relations between absolute fractions of habitat encounter, kin avoidance, and colonization (at optimal dispersal strategies) and landscape parameters patch size and patch turnover. The optimal values of *μ* for these landscapes, derived from pairwise invasibility plots are shown in the bottom right panel.

**
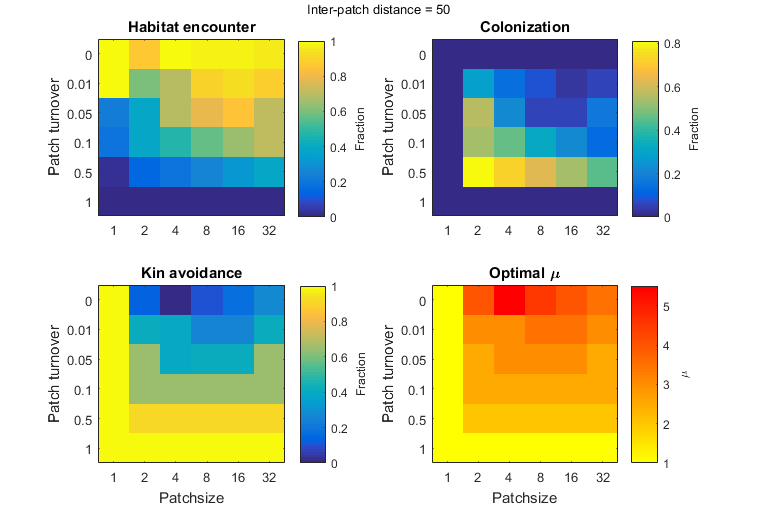
**

**Figure S5b**. Relations between normalized fractions of habitat encounter, kin avoidance, and colonization (at optimal dispersal strategies) and landscape parameters patch size and patch turnover. The optimal values of *μ* for these landscapes, derived from pairwise invasibility plots are shown in the bottom right panel.
